# Walk in our shoes: older homeless women's perceptions of the challenges of navigating streets and shelter life

**DOI:** 10.3389/fgwh.2026.1697164

**Published:** 2026-04-17

**Authors:** Judith G. Gonyea, Kelly Melekis

**Affiliations:** 1 School of Social Work and Center for Innovation in Social Sciences, Boston University, Boston, MA, United States; 2 College of Education and Social Services, University of Vermont, Burlington, VT, United States

**Keywords:** aging, emergency housing, homelessness, poverty, precarity, shelters, trauma, women

## Abstract

**Introduction:**

Older women experiencing homelessness (OWEH) have been a hidden or invisible population; however, with rising numbers globally, they are gaining attention. Our study examines what it means to be a woman in her 50s who is struggling financially and precariously housed in the United States. Although in their 50s, these women often experience accelerated aging and exhibit chronic health conditions comparable to those of housed women in their 70s and 80s. Lacking access to both public old-age benefits and family support, they often fall between the cracks of the nation's safety net system. While homelessness among women of reproductive age, including the role of gender-based violence in increasing their vulnerability, is now well documented, studies on the unique challenges faced by OWEH remain relatively limited.

**Research objective:**

This study explores how daily life on the streets and in emergency housing shelters, a system historically designed for men (particularly younger men), affects the well-being of OWEH and their journey toward stable housing. The aim was to describe, from the perspectives of OWEH, how shelter environment, policies, and practices (including continual displacement to the streets) shape their daily lives, well-being, and pathways to stable housing. Of particular interest was gaining a deeper understanding of how navigating the traumas of homelessness and shelter living affects individuals' sense of dignity, self-worth, adaptive resources, and resilience.

**Methods:**

This qualitative study involved 15 semistructured, private individual interviews, each lasting about 60 min, conducted by an MSW social worker. Eligibility criteria included being homeless for at least 1 month, being in one’s 50s, and being able to take part in an English-language interview. Using NVivo (qualitative research software), the interview audiotape transcripts were coded and analyzed using a multistep interpretative phenomenological approach to enable exploration of how OWEH make sense of what is happening to them (or the “lived experience') and their views (or “meaning making') of life and these experiences.

**Results:**

Trauma was a universal experience, and almost all participants were coping with significant physical and/or mental health issues. Overall, participants perceived that shelter management and staff failed to fully understand the challenges women faced in rebuilding their lives, especially the interconnectedness of health struggles, societal bias against older women, and the social prejudice of people experiencing homelessness. Five superordinate themes were identified, highlighting how shelter physical and social environments contribute to OWEH's daily struggles and sense of disempowerment: (a) dehumanizing and stigmatizing treatment; (b) unsafe surroundings and hypervigilance; (c) harsh living conditions and declining physical and emotional health; (d) disempowering situations and loss of control; and (e) an absence of normalcy and stability.

**Conclusion:**

This study contributes to our understanding of how emergency housing shelters create or exacerbate the challenges faced by OWEH and often result in disempowering. The findings suggest the importance of transforming both the physical and social environments of shelters, using trauma- and aging-informed approaches, to better support this growing population of OWEH in their pathway to stable housing.

## Introduction

Older women experiencing homelessness (OWEH) have been a somewhat hidden population, given their avoidance of the formal shelter system and greater reliance on family or friends, rough sleeping, and/or remaining in unsafe living arrangements, often under the threat of violence. Their spatial invisibility (or absence from public view) has contributed to their invisibility in policy, practice, and research focused on homelessness prevention and intervention. However, as their numbers rise globally, OWEH are gaining public attention. Mirroring the aging of the U.S. population, the median age of the single (or solo) U.S. homeless adult population is now 50 years—a steep climb from a median age of 35 in 1990 (1, 2). Approximately one in every five persons experiencing homelessness on a single night in 2024 was aged 55 years or older (3). In addition, although long a refuge primarily for men, women now represent approximately 31% of single homeless persons seeking an emergency housing shelter bed each night in the United States (3). Despite their growing presence, research exploring the intersection of age and gender in the homelessness experience remains relatively limited. This study builds on our previous research exploring precariously housed older women's exposure to multiple forms of trauma, such as stigmatization, victimization, and violence (4). In this qualitative study, we focus specifically on women in their 50s experiencing homelessness, a decision based on two factors. First, in the United States, women in this age group often face distinct challenges, as they are neither eligible for federal and/or state programs targeting families with children, since they generally do not have minor-age children, nor are they sufficiently old (typically defined as age 62 and older) to qualify for old-age benefits. As “tweeners” between these two targeted populations, these women face a greater risk of falling between the cracks in the nation's safety net system. The stark reality is that they typically have limited access to public resources aimed at preventing and/or ending their homelessness.

Second, the vast majority of the chronically homeless older adult population in the United States is between 50 and 64 years of age; only about 5% are 65 and older (3). The reality is that persons experiencing chronic homelessness are less likely to reach the traditional marker of old age (defined as age 65) compared with the general population. Thus, the lower age threshold for “older homelessness” is generally set at 50. This is because, due to poor nutrition and harsh living conditions, persons who are chronically homeless experience accelerated aging and present with chronic health conditions and functional limitations similar to those of housed individuals in their 70s and 80s (5–7). In this study, our exploration of what it means to be a female in her 50s who is struggling financially and precariously housed is focused on gaining a deeper understanding of how daily life on the streets and in emergency housing shelters, a system historically designed for men (particularly younger men), affect OWEH’s well-being and their journey toward stable housing. The goal of interpretative phenomenological research is to describe the meaning of an experience—both in terms of what was experienced and how it was experienced. By listening to women's accounts of their journey into and navigation of homelessness, we sought to gain insights into how shelter environment, policies, and practices (including continual displacement to the streets) affect their sense of dignity, self-worth, adaptive resources, and resilience. In essence, we explored how women experiencing the trauma of homelessness strive to maintain a positive sense of self, preserve some aspects of “normalcy' in their lives, and sustain a feeling of hopefulness for their future.

Emergency housing shelters are widely recognized as a vital community resource, providing an immediate place for individuals and families experiencing homelessness to get off the streets and access basic resources (i.e., a bed, food, showers). Since their origin, emergency shelters have been viewed as temporary short-term solutions, offering a first stepping stone toward rebuilding lives and transitioning to more permanent housing. Emergency housing shelters were never envisioned (or designed) for long-term residence. However, research suggests that the transition from emergency shelters to transitional or permanent housing can be particularly challenging for some persons (8, 9). A recent quantitative study, for example, revealed that longer shelter stays were associated with being female, older, a member of a racialized minority group, and having a disability (9). The study also found that “in most age groups, the length of homeless shelter stays for women remains longer” than for men (9). This qualitative study offered the opportunity to explore in greater depth how older women, many of whom are women of color and/or living with a disability, navigate homelessness and the shelter experience.

### Background

Persons experiencing homelessness have long been categorized into a singular, stigmatizing identity of “the homeless.” However, although the fundamental cause of homelessness is poverty and the lack of affordable housing, it is not a monolithic population. Due to historical and ongoing discrimination, people experiencing homelessness often have multiple marginalized social identities (e.g., race, gender, disability). The United Nations (10) has identified homelessness as one of the cruelest manifestations of poverty, discrimination, and inequality. In the United States, the impact of historic and ongoing structural racism on housing stability is reflected in national data showing that most racialized minority groups experience homelessness at higher rates than White people. For instance, Black Americans comprise 13% of the general population but account for more 40% of the nation's homeless population (11). Across racial groups, homeless families in emergency shelters are almost always headed by women (3, 7).

Research suggests two predominant pathways into homelessness in later life (4, 5, 7). The first involves adults with a history of stable housing who experienced homelessness for the first time in older age. These persons' narratives often center on certain trigger events or a series of recent misfortunes that upended their lives. The second pathway includes persons with a history of periods of homelessness in their earlier adulthood who simply “age into' the older homeless adult category. These individuals often speak of the myriad problems that prevented them from breaking this cycle of unstable housing. They report having faced negative life experiences in their young or mid-adult years (e.g., mental health issues, substance use, trauma), as well as less frequent attainment of typical life-course milestones (e.g., educational degrees, marriage, steady employment).

Research also suggests that women's pathways into and experiences of homelessness differ from those of men. The gendered nature of life experiences, historical discrimination, marginalization, and disempowerment of women, as well as greater exposure to sexual harassment and abuse, contribute to a heightened risk of financial insecurity and housing precarity among women. When women become homeless, they continue to face these heightened vulnerabilities to a greater extent than their male counterparts (12, 13).

## Methods

This study was part of a qualitative research project on older women experiencing homelessness in an urban Northeastern U.S. city and was approved by the Boston University Institutional Review Board. A purposive sampling approach was used, with three eligibility criteria: participants had to have been homeless for at least 1 month, be currently in their 50s, and be able to participate in an English-language interview. We are indebted to the generous support of a local women's day shelter, which allowed us to recruit women via a posted flyer and provided a private office space for conducting the interviews. The flyer explained that: (1) the purpose of the study was to gain a better understanding of the challenges of homelessness among women in their 50s, (2) participation would involve a single private interview lasting 50–60 min, (3) questions would focus on their own journey to homelessness and their views on resources needed to help women rebuild their lives, and (4) each participant would receive $40 as a “thank you.”

Informed consent, including permission to record, was obtained prior to the interview. A total of 15 semistructured interviews, each averaging approximately 60 min, were conducted by MSW social workers. The interview guide included five core questions, asking women to describe: (1) the pathway and nature of their current homelessness, as well as any prior episodes, (2) the personal challenges, needs, and problems they currently face in daily lives, (3) the most helpful resources, sources of support, and/or services they have received while coping with and navigating living without a home, (4) the least helpful or missing resources, sources of support, and/or services while coping and navigating with homelessness, and (5) their sense of hopefulness regarding their personal future.

Each interview was transcribed in its entirety; pseudonyms (selected by the interviewee or interviewer) were used to protect anonymity. Using transcribed interviews, an interpretative phenomenological analysis (IPA) approach was applied to explore how individuals make sense of significant life experiences. IPA combines phenomenology (the study of lived experiences) and hermeneutics (interpretation), acknowledging that researchers interpret participants' interpretations (a process known as double hermeneutics) of complex and often emotionally laden life events, such as homelessness (14, 15). A preliminary step in our IPA analysis involved becoming familiar with the data through repeated reading of the transcripts, allowing for deep immersion. NVivo qualitative research software was then used for the data coding process, including initial open line coding to identify descriptive and conceptual themes within each case. The next step involved grouping these initial codes into broader themes for each participant. This was followed by cross-case analysis to identify patterns, similarities, and differences across participants, leading to the development of second-order emerging themes. This funneling process led to the generation of superordinate themes. Given the interpretive phenomenological nature of the study (aimed at understanding the lived experiences of OWEH and how they make meaning of these experiences) and the value of “displaying the comments of those being researched in their voices” (16), longer quotes are used to provide broader explanations of concepts.

### Participant characteristics

 Table 1 presents the demographic characteristics of the 15 women, who ranged in age from 50 to 59 years old. The majority of participants identified as women of color: nine as “Black' or “African American,” five as “White,” and one as “Black and Native American.” In terms of education, nine women had completed high school, four held bachelor's degrees, one had attended but not graduated from high school, and one held a master's degree. Almost all participants self-identified as heterosexual; one identified as bisexual, and another spoke of being asexual. Regarding marital status, nine women reported being “single and never married,” five were “single and divorced' and one was “separated and seeking a divorce.” Except for two women, all others had children. Nine women had adult children (including one whose son was living on the male side of the same shelter), two had both adult and teenage children (the latter not in their custody), and two had teenager children in state custody.

**Table 1:** Participant characteristics (*n* = 15).

Variable	*f*	%
Racial identity		
Black/African American	9	60
Black and Native American	1	7
White/Caucasian	5	33
Sexual orientation		
Heterosexual	13	87
Bisexual	1	7
Asexual	1	7
Education		
Less than a high school graduate	1	7
High school graduate	9	60
Bachelor's degree	4	27
Master's degree	1	7
Marital status		
Single and never married	9	60
Single and divorced	5	33
Separated, seeking a divorce	1	7
Children		
None	2	13
Adult children only	9	60
Teenage children only	2	13
Adult and teenage children	2	13
Pathway into homelessness		
Homeless for the first time after age 50	6	40
Homelessness began in earlier adulthood and never gained stable housing	4	27
Homelessness began in early adulthood, gained stable housing for years, yet became homeless again after age 50	5	33

The women had either roots in, or a connection to, the city or its greater metropolitan area. Almost all were born or raised in the city or its surrounding communities and/or had family members living close by. Arriving in the United States, one woman, an undocumented immigrant, spoke of specifically choosing this destination because two of her aunts lived there. The only exception was a woman who had relocated from another region of the state to create greater geographic distance from an abusive ex-husband.

Most of the interviewed women were coping with multiple chronic health conditions, including both physical and mental health issues. Only two participants described themselves as “healthy' and reported not taking any medications. Cited health problems included diabetes, cancer, heart disease, high blood pressure, arthritis, anxiety disorders, panic attacks, depression, bipolar disorder, and post-traumatic stress disorder due to domestic violence and/or sexual assault. Several women reported significant physical pain, mobility challenges, and dental problems. The women framed their health by speaking about current and past hardships and traumas.

Their pathways into homelessness revealed that many were “living on the edge' caught in a downward spiral, and “struggling hand to mouth' before losing their stable housing. In our study, we identified three distinctive pathways. As shown in Table 1, of the 15 women, six experienced homelessness for the first time after the age of 50; five had a prior incident of homelessness in their 30s or early 40s, regained stable housing for several years, but later became homeless again in their late 50s; and the remaining four became homeless in their 30s and 40s and never regained stable housing, simply aging into “older homelessness.” Eviction was stated as the primary reason for homelessness for most participants; one woman cited “incarceration” as the main factor, while another cited family conflict. However, the stories of their evictions often invoked multiple complex issues such as health decline, job loss, domestic violence, family discord, gang violence, and landlord greed and disputes.

In addition, for nearly all women, their journeys into homelessness involved the use of multiple sheltering options and frequent displacement, including house surfing with family and friends, staying in emergency housing shelters, accessing domestic violence shelters, and rough sleeping in parks, woods, and/or bus and train stations. The women also reported typically using multiple emergency housing shelters. Only one woman, who became homeless for the first time in her 50s, relied on a single emergency housing shelter as her refuge from the streets.

## Results

The interrelationship between physical place, social connections, and identity is central to understanding the profound negative impacts of homelessness on older women's lives (17). Rowe and Wolch noted that, for most homed individuals, “our social networks and daily paths create a powerful sense of time-space continuity which in turns molds our individual identity and self-esteem.” (17) In contrast, they stressed that “homelessness creates time-space discontinuity and that this struggle to survive in a degraded and threatening environment can alter permanent identity and have an impact on self-esteem.” (17) The women's lives on the streets and in the shelters were intertwined, as each morning they faced compulsory displacement from the shelter into the community**.** Below, we present the five emergent superordinate themes, which highlight aspects of the shelter environment, both physical (or spatial) and social (or relational) conditions, that the women described as further intensifying their daily struggles and disempowerment. For each theme, quotes are used to both elevate the women's voices and provide a broader explanation of the superordinate themes.

### Theme 1: dehumanizing and stigmatizing treatment

Shelters were viewed as dehumanizing places that further stigmatize OWEH and diminish their sense of self-worth. The women often rallied against and resisted the stereotypical and negative identity of “homeless.”

Indeed, the interviewed women were united in their frustration and anger about what they perceived as the shelter staff's failure to see them as unique individuals facing hard times and instead cast them into a singular, one-dimensional identity of homeless, often imbued with stigmatizing representations of personal inadequacy or moral weakness. The following comment captures well the interviewed women's sentiments:

I think that they [shelter staff] should see the homeless as a human being. A homeless person is not a number … They always give us bed numbers, call us by bed numbers … We are all dumped together as the homeless, but we are people. We are individuals with different belief systems, different cultures, different backgrounds… Staff just think they're dealing with numbers, and they think everybody who comes in there is a drug addict or a prostitute…The most painful thing about the homeless situation is that we are all dumped in one big, rotten pot—homelessness.

However, the women differed in regard to their personal acceptance of this stigmatizing identity. Those rejecting the label of homeless offered alternative self-descriptions or definitions of their status, such as by saying:

-I wouldn't really say homeless; I would say without a house right now.

-1 haven't embraced homelessness fully, it's a label or a choice. It's a state of mind.

-No, I consider this a temporary situation. It is not my life, and I am determined to regain my life. So, I guess I just don't.

Others clearly identified themselves as being among the ranks of homeless persons; examples included the following:

-I am homeless; I have already accepted that fact.

-On December 31st 20xx, I received a court ordered eviction notice; that is when I realized that I was homeless.

-The last time I had my own place was right before I was incarcerated, over 20 years ago. I've been homeless for about 20 years.

Regardless of whether the women viewed themselves as homeless, all reported having this negative identity or label imposed upon them. Trying to hide her homelessness each day as she left the shelter and entered the streets, this woman explained,

I don't want people to know I'm homeless because they lump you into a category of being a prostitute, a drug addict, a drug dealer, alcoholic, severely mentally ill or mentally retarded, incapable, and less than human. The attitude is prevalent in our society; there is real stigma attached.

### Theme 2: dangerous surroundings and hypervigilance

Shelters are dangerous places in which individuals must constantly be on guard to protect themselves from harm.

Women often raised concerns that the shelters and their surrounding “rough' or “sketchy' neighborhoods were dangerous places. They shared their worries and/or experiences of being targeted for harassment and verbal abuse, physical violence, sexual assault, and/or theft of personal belongings while on the streets. Multiple references were made by the women of shelter life being like “prison life' particularly in terms of threats to physical safety. For OWEH, especially those encountering homelessness for the first time in their lives, shelter life was often described as a “culture shock,”

-You know, because the first two months in that shelter is the hardest. Let me tell you, it's like a little prison. It's hardcore! You've got bullies, you got all these people bumping into you, pushing you around. It's tough in a shelter. It's very territorial… I didn't get a locker because a lot of drama and fights break out at where the lockers are.

-Sometimes I feel like it's a mini-prison because of some of the things that go on—off the record, you know. But I'm not going to let the attitude of the staff members and some of these people who have been there for a long time and feel entitled to the place, gang up to throw people like me out. It is very territorial at the homeless shelter.

Many of the women interviewed reported being hypervigilant; that is, constantly scanning the shelter environment and/or always remaining on guard for potential imminent threats or dangers. Identified coping strategies to escape bullying from coresidents, disrespectful staff, and/or the chaotic atmosphere of shelter life included moving between different emergency housing shelters, seeking temporary refuge in the woods or public spaces such as bus or train stations, and sometimes just keeping to themselves in the shelter. When asked why she alternated between spending nights at a bus station and in the shelter, this woman responded,

Have you ever been to the shelters? It's not a good place. I feel safer at the bus station.

Because people that are in the shelters are really kind of gangsters, street people..

Um, I sleep with my bag under my pillow.

Another participant spoke of some shelter residents as “almost a gang, cliquish…vicious…I think they feel that the newbies are interloping on their space.” Offering a similar perspective, another woman noted, “in the beginning, I was bullied more than I am now. I was fragile, uh, more fragile than I am now because I had just come off that [domestic violence] abuse.” As this woman shared,

A lot of thought process goes on to, uh, coping in this environment…People have trouble with personal space…It gets better the longer you're here…you get to know people's quirks…you learn quickly to give some a lot of space…don't even make eye contact with them.

Several women emphasized their greater vulnerability due to their older age, particularly in terms of the aggressive and threatening behaviors of younger women (who were often described as “bigger and stronger') toward them.

-These young girls are coming in and they're pretty rude and pretty mean girls. We've been called the old B's …Yeah. A lot of threatening going on. They really need to have their own space, or you know, our age group we really need to have our own space. I mean, these girls, you know, are threatening to um physically assault you. I mean, it's like every like day.

Some participants also raised being troubled by the staff's failure to address bullying and conflicts among the residents. In addition, negative attitudes or behaviors from some staff members toward shelter clients were perceived as contributing to the sense of danger within the facility.

-People like to say things about me or they'll pick a fight…Every time I go to a staff, they don't do anything.

-I don't like fighting. They, uh, argue over seats in there and, you know, put their bags in the chair, save a seat. They do what they want to do, and staff don't do nothing, okay, but just stand there. Stand there, see it's going on, and definitely do nothing.

-You know, the shelter in [place] is horrible. The people, the management need to quit; their attitude to people is horrible. They probably are over-saturated with their jobs, and they just need to give it up and go home. Because they've left people very injured, they're very mean to people.

### Theme 3: harsh living conditions and declining health

Shelters are “bare-bones' places that offer limited physical or emotional comfort; a misalignment exists between the health conditions and needs of OWEH and the shelter environment and resources. Indeed, many OWEH found the shelter's harsh living conditions difficult, uncomfortable, stressful, and arduous. The lack of supportive resources further amplified this mismatch between the their capabilities and surroundings, contributing to chronic exhaustion and further deterioration of their physical and emotional health.

The interviewed women often raised the “rough conditions' of life in the shelter. A common concern was the sleeping arrangements, given their age and associated health problems.

-I don't see any types of, you know um, consideration for older women, especially in terms of bunk beds…It's all first come, first served…you get whatever bed is there. There are no pillows. Sometimes they run out and you are given just one bed sheet and no blanket…it can be challenging…it's a very down to the bones place.

-If the staff think you are well-abled, you get a top bunk regardless of your age… You know, I can do a top bunk, but I don't like a top bunk. It's hard, I have to get up frequently during the night to go to the bathroom.

-At times, I had to either sleep on a mat on the floor or they have like these cots. How can I explain it? They're cloth cots…one on top and one on the bottom connected by like metal rods. Now, if somebody is like a really heavy woman, and you are sleeping on the bottom part, it's like, it curves into your body. Many times, I have bumped my head trying to get out. I've had problems even getting off of it and getting up off the ground. I have bad leg cramps.

The total lack of privacy in shelters was a difficult adjustment for many participants. As one woman emphasized, “I am sleeping with 110 roommates; nothing in my life prepared me for this!” The women also viewed the shelters as “bare-bones' environments, characterized by “a lack of feminine products,” “limited shower time,” “unhealthy food options,” and “limited storage lockers.” The women spoke, for instance, of the challenges of consistently traveling with their possessions, whether the weight of baggage or its marking them as homeless.

-The first sign of living in a shelter is you hold on to things. I'm used to holding my bag; I got all my IDs and stuff in it. Inside my bag is a box, it has all my personal diaries and stuff. So, I always hold my bag—that's my life actually!

- So normally, I have like a small kind of carry-on suitcase. But wheeling that across town, it's too much for me. Also, I'm too embarrassed. I have complete and total shame … I'm out of place, like normal people don't walk around all day with a suitcase. They know you're homeless.

Despite the shelter being bare-bones and having limited resources, some women identified the value of receiving emotional support from staff.

-I told one of the staff I'm having a panic attack, and I've already taken my three pills for the day and it's not working. She asked, what do you feel like doing and I says I feel like beating someone up. She asked, what can we do and. I says, find someone that can talk to me; usually if I talk to someone 1 calm down. They did and I felt like um, like a rush–no more panic…I says thank you for helping me get rid of my panic attack. Now, I sit here every day, just in case.

### Theme 4: disempowering situations and loss of control

In shelters, OWEH feel a loss of a sense of control, self-determination, freedom, and independence; shelter rules, protocols, and practices are often disempowering.

The women described how shelters' “rigid' and “strict' rules, protocols, and practices exacerbated feelings of a lack of control over their lives and the sense of being powerless. Several women identified specific shelters they avoided, or used only as a last resort, due to perceived overly restrictive rules. Many women also expressed frustration with staff interactions, citing judgmental attitudes or unequal treatment. Women also spoke of some staff having “favorites' who were treated better, while some raised concerns about “racism' in the enforcement of rules.

- Well, I tried to get into other programs. And the reason why I've come back is because .. I mean, one [place] was really, really horrible… It was terrible. I mean, we were treated like we were in prison. Like, we didn't have any rights whatsoever.

-Because I felt that the women who were running it [the shelter] were just like really kind of vindictive and kind of um, um .. yeah. I didn't see .. you know, they didn't accept any kind of excuses. It was run sort of like the army.

Flexibility, the ability to have some freedom and choice, was a desired option that the women found difficult to find.

- At [place], we have a lot of flexibility. Um, when you come in, you know, you can do whatever you want to do…Some people go directly to bed, and they just sleep for 12 h. Other people, we have like little lights by our beds. You're allowed to keep it on until about 11:00 or 11:30…I think if you, if you give people the benefit of the doubt, then they are gonna be humane. They're going to like, uh, um you know, be kind of considerate and things like that…This program, you know, they don't have a lot to offer you in terms of you know…the physical things…But you feel humanity and that's why I've been coming back there.

For many women, the sense of powerlessness began with the process of securing a bed for the night. The protocol, which typically involved being assigned a number and participating in a lottery for a slot, quickly introduced them to the regimented nature of shelter life.

-They often open the shelter doors at 3 p.m…Some of us, I have gone to line up from 12:30, 1:00, or 1:30 because you just want to find a place to stand somewhere in the shade or something else. Or you arrive early because you want to be sure to get a bed or you have nowhere else to be…Sometimes we stand for hours…But there's no guarantee that at 3:00 it will even open. There's been instances where they said there's no security or something, so we'd still be standing there until 3:30, and sometimes the weather is raining or something, but they say that “Well, they are not obligated to open the door for us until it's open.”

Once admitted, shelter policies and protocols continue to reinforce OWEH's sense of lack of freedom, control, and power throughout their stay. Describing her mornings, one woman spoke of “knowing the drill of getting up and getting out' with no control over when or how her day begins. She also shared the daily stress of waking up to the unpredictability of living in a public space.

- I get up at 6 am. Lights up. Some staff are rude; some are nice. Some give you extra time; some put on lights earlier than they're supposed to. Some residents are cruel; some are very respectful. So, every day is different, and it throws me off my routine. You go to your locker… you have to strip your bed of all the sheets. You deposit your sheets and scrubs into bins and then get yourself dressed and go down. There's breakfast from 6 to 7 am; the building closes at 8:30 a.m.

Faced with restrictive schedules for different activities (i.e., showers, meals, TV viewing, bedtime) and strict guidelines for behaviors (i.e., wearing scrubs, shower length, possessions), the interviewed women shared, often with a sense of pride, their agency and resourcefulness in circumventing or overcoming many such obstacles. For example, speaking about the importance of her possessions, this OWEH explained how she “gets around' the rule allowing only one bag of a specified size in the sleeping-area lockers.

-So, in order to pass customs, I carry my stuff in one right sized bag to [sleeping area,], then I break it into three bags-my clothes, my handbag, and my treasures. This [treasures] will always, this sleeps with me. And you're not supposed to sleep with anything in the bed. It's supposed to literally be in the bag. I put it in my scrubs and so they don't, they've never caught me… If you live at [place], you better have a routine!

### Theme 5: absence of normalcy and stability

Focused on pursuing some normalcy and stability in their lives, OWEH often expressed aspirations “for a home” to gain a sense of permanence, comfort, and continuity. For instance, faced with the unsettling experience of sleeping alongside multiple strangers, to elicit a feeling of “being at home,” this woman's nightly ritual was to watch cat videos on her cell phone before falling asleep, until it was stolen.

-You're going to laugh at me. I used to look at cat videos on my phone. It helps me because I think that it evokes, um, positive—mushy-gushy feelings…It brings me back to a gentler space and time, and I can-I can pretty much imagine that I'm in my own bed and my own home with my own pillows. With my cats and dogs…So it's a self-help.

Many women's efforts to hold onto normalcy in their lives focused on maintaining their physical appearance, including hygiene, hair, makeup, and clean clothing. Similar to the findings by Preece and colleagues (18) on persons experiencing continual housing displacement, we found that such “self-care' helped individuals maintain a connection to their non-homeless identity, including the ability to pass as “housed.” These daily practices of “keeping up one's appearance” also represented one of the few areas over which the women felt a sense of control. In listening to women speak about their efforts, we heard both pride in their resourcefulness and fatigue from managing this care within constrained environments. With no laundry facilities in her shelter, for instance, this woman spoke of how she engineered washing her clothes.

-I hand-wash a lot of things and you have to be very tricky about it. They don't allow it in any of the shelters… Um, I've asked for special circumstances. Like once I spilled sauce on a blouse, “So [I asked] can I please wash out the stain?” And then while you're at it, you just do the whole thing.

Other strategies described by women included accessing laundry facilities at day shelters, paying for storage lockers, and relying on housed family members or friends. As this woman shared,

-You know, it's everything to have these places [day shelters]. You have to be very strategic when you're homeless. Very strategic. …You learn skills that you never knew you'd learn how…I store my clean clothes there…They give you a bin. …I'll just get my clean clothes, and I can wash clothes if I want to.

Daily displacement to the streets was tiring and wearing on all women. In reality, they must seek out the most basic goods that others access within their homes, including food, toilets, clothing, laundry facilities, computers, and Wi-Fi/internet. For many interviewed women coping with serious health problems, a significant amount of travel is devoted to medical appointments. Travel is also about simply finding or claiming a spot to be (until the shelter reopens)—whether a park bench, a street corner, public library, or a women's resource center. The women often reported that their daily travels, almost all by foot or public transportation, were challenging, frustrating, and tiring.

-I usually walk to my appointments because I'm indigent … It's a long process to walk. I just walk everywhere. I have to take frequent breaks because of my medical condition. …I spend an awful lot of time at doctors' offices…I'm menopausal… I have bone problems, I have cancer… I have lumps in my breast …I know that I could be much more proactive finding housing, and I'm falling short. I'm consumed with the domestic violence in my family, with worry about my health and just navigating—just getting through the day—it consumes me.

All but one woman spoke of being hopeful of eventually finding safe, secure, affordable housing; however, on a daily basis, they often felt “stuck in place' and/or frustrated by the “paralyzing slow process.” This woman's remarks on the everyday challenges of not having housing capture this type of sentiment well.

-It's, just the, uh, the lack of stability. Just having some place where you can, you know you can go every day. Not to have to worry about a certain time to eat, a certain time to go upstairs to bed, you know what I'm saying? …A certain time to watch TV. I want to be comfortable..You know what I'm saying? And I don't want it to take forever. Which, you know, I have patience. But, you know, I'm not young. I'm not no spring chicken. You know what I'm saying? Right now, I need my life to be where I have my place, where I can be comfortable, and do the things that I like to do in life now. Oh, it's driving me crazy. It really is. It's driving me crazy …You know, it's like, I'm lost. It's like, I'm lost…

## Discussion

Although housing market pressures, unaffordability, and homelessness in U.S. urban cites are clearly not new phenomena, the rapidly growing number of older adults within the nation's adult homeless population (associated with the aging of the baby boomer generation, persons born between 1946 and 1964) is a rising concern. A 2019 study (2) estimated that the U.S. older homeless population will triple by 2030 and warned that this may lead to a surge in public costs associated with healthcare and shelter needs. Older single women (whether widowed, divorced, or never married) are at greater risk of facing poverty in later age compared with their male counterparts. Given the close relationship between poverty and homelessness, it is likely that their numbers within the emergency housing shelter system will continue to grow.

A key question is whether the emergency housing shelter system will adapt to the shifting demographics of its service users, and, if so, how it can better support older homeless women in rebuilding their lives and transitioning to stable housing. This study sought to inform this transformation by giving voice to older women currently engaged with the shelter system to identify how the shelter environment affects their well-being and their journey toward stable housing.

OWEH valued shelters for offering the refuge of a bed, a meal, and a shower. However, the findings of our study also revealed that they perceived shelters as posing multiple threats to their physical and emotional well-being, as reflected in five emergent themes of OWEH perceptions on how the shelter environment contributes to their daily struggles: (1) dehumanizing and stigmatizing treatment, (2) dangerous surroundings and hypervigilance, (3) harsh living conditions and declining health, (4) disempowering situations and loss of control, and (5) an absence of normalcy and stability. These phenomena (or experiences) were intertwined in the OWEH's lives, both on the streets and within shelters, often cascading and overwhelming their efforts to move forward.

In their interviews, women expressed their gratitude to certain staff members who were supportive, kind, and responsive to their needs, whether by just listening to their daily challenges or successes, offering comfort, or demonstrating flexibility in applying shelter rules. However, the general sentiment was that shelter management and staff simply did not fully understand the significant hurdles they, as women in their 50s, experienced in the quest to rebuild their lives, especially the intertwining of their health struggles (i.e., pain, mobility challenges, medication and adaptive equipment needs, and fatigue), societal bias toward older women (i.e., being invisible or judged incapable), and societal prejudice of people experiencing homelessness (i.e., being labeled as deviant, weak, and/or deficient).

A limitation of this study is its relatively small sample size of older homeless women, all of whom were living in a single urban community in a northeastern U.S. state. In addition, participation was limited to English-speaking women. Finally, as a stigmatized and marginalized population, some OWEH (perhaps those who are more disenfranchised) may be less likely to volunteer or self-select for participation in a study on homelessness. Women who are willing to share their homelessness story with a research team may differ from those who choose not to engage in such an activity.

It is important to highlight that, consistent with prior studies (19–22) showing that 90%–100% of homeless women have endured traumatic events (often multiple traumas), trauma was almost universal among the OWEH interviewed in this study. Indeed, the 35%–80% prevalence rate of lifetime PTSD across homeless women studies far exceeds the estimate that 8%–12% women in the U.S. population will experience PTSD at some point in their life (23–26). Thus, our core finding—that the physical and social environments of shelter' can be re-traumatizing for OWEH—is a significant concern. These environments often reinforce women's distrust of people, fears of safety, and feelings of disempowerment and despair.

For many OWEH, hypervigilance (a constant state of high alert, often a physiological consequence of trauma) is both a survival mechanism and an impediment to long-term stability. Indeed, our study findings reveal that both the physical structure and authoritarian practices in many shelters may exacerbate, rather than alleviate, hypervigilance. Entering a large, loud, or chaotic shelter, as well as witnessing or experiencing verbal threats, physical violence, or substance use among shelter residents, can lead to fear and trigger anxiety about personal safety. This may, in fact, be as traumatizing as the situations OWEH fled or life on the streets. Our study also highlights how strict rules and limited autonomy can reinforce feelings of stigma and control, rather than support. The rigid rules led some OWEH to describe shelters as “prison-like” and to express feelings of “being trapped.” Rather than empowering women, such authoritarian practices may deepen their sense of disempowerment, undermining their agency and ability to make decisions that could improve their situations.

It is now widely recognized that stressful and traumatic events not only predispose women to homelessness but that homelessness itself also places women at risk for experiencing traumatic events. This study underscores that this vicious cycle is further worsened by acute and chronic physical and mental health challenges (including accelerated aging and geriatric conditions), which are commonly observed among women aged 50 and older who have experienced homelessness.

Emerging research also suggests that the physical and cultural environments of the homeless service system do not effectively support either shelter users or staff. Recent studies have also reported high rates of stress, depression, and post-traumatic stress disorder symptoms among homeless service staff (27). A paradigm shift is emerging regarding the incorporation of trauma-informed care (TIC), a person-centered approach in homelessness programs and services, which acknowledges the impact of the myriad of traumas lived through by persons experiencing homelessness and, by focusing on individual needs and preferences, empowers them to make decisions about their own lives (28). In essence, it represents a fundamental shift from asking “What is wrong with you?” to “What happened to you?” (29). In 2014, Substance Abuse Mental Health Services Administration (SAMHSA) published a paper on the concept of trauma and guidance for a trauma-informed approach (TIA) to create “a shared understanding of these concepts that would be acceptable and appropriate across an array of service systems and stakeholder groups (30).” SAMHSA outlined six key principles of a TIA (safety; trustworthiness and transparency; collaboration and mutuality; empowerment, voice, and choice; and cultural, historical, and gender issues). SAMHSA also emphasized that implementating a TIA in organizations would require a multilevel response, encompassing domains such as governance and leadership, policy, physical environment, workforce development and training, as well as quality control, monitoring, and evaluation. Among organizations serving homeless women in the United States, we are witnessing a shift toward TIAs; however, these efforts do not typical involve a holistic, multilevel approach. In addition, there remains a scarcity of rigorous evidence-based outcome studies evaluating TIA/TIC for women experiencing homelessness, as well as a lack of TIA research focused on OWEH (29).

As the field advances, we advocate for a gender- and age-specific focus. With the dramatic growth of the OWEH population, women experiencing homelessness, housing and homeless organizations must incorporate approaches that are both trauma-informed and responsive to aging/geriatric needs. To achieve this goal, OWEH must no longer be an invisible or absent population but should be included in the development, implementation, and evaluation of these interventions, given their heightened risk of mental health issues. There is a need for more qualitative research, such as in-depth interviews, to capture lived experiences of older women who have experienced precarious housing and to understand their perspectives. Finally, there is also a need for mixed-methods studies to identify the impacts of TIAs on OWEH, not only in the immediate postintervention period but also in terms of long-term healing and stability.

## Data Availability

The datasets presented in this article are not readily available because the interview audiotapes have identifiable information and are therefore not available. Requests to access the datasets should be directed to jgonyea@bu.edu.
